# Empirical research on the friction behavior of O-rings in hydraulic cylinders

**DOI:** 10.1371/journal.pone.0280815

**Published:** 2023-01-23

**Authors:** Zhen Qin, Yu-Ting Wu, Lei He, Xiang Gao, Sung-Ki Lyu

**Affiliations:** 1 School of Mechanical Engineering, Shandong University of Technology, Zibo, Shandong, China; 2 School of Transportation and Vehicle Engineering, Shandong University of Technology, Zibo, Shandong, China; 3 Tianrun Industrial Technology Co., Ltd., Weihai, Shandong, China; 4 School of Mechanical and Aerospace Engineering, Gyeongsang National University, Jinju-si, Gyeongsangnam-do, Korea; China University of Mining and Technology, CHINA

## Abstract

Mechanical products are becoming more diversified with the continuous development of precision processing and materials technologies. The friction force generated by the O-ring seal in a hydraulic cylinder was once considered redundant. However, its utilization has recently been proposed. The hardness of the O-ring and the inner diameter of its groove directly affect the normal pressure between the O-ring and the inner wall of the cylinder, thereby affecting the friction behavior. In order to explore this friction behavior, a strain-based friction force measurement system is developed in this study, and the steady-state and dynamic friction values under different working conditions are studied and discussed in depth. This research on the friction behavior in the cylinder provides a theoretical basis for more convenient design and utilization of the friction force generated between the O-ring and the inner wall of the cylinder.

## Introduction

The hydraulic cylinder is a key element of the hydraulic system that is responsible for the energy conversions between hydraulic and mechanical forms [1, 2]. Hydraulic actuation and viscous hydraulic vibration energy absorption are the two most common applications of hydraulic cylinders. The hydraulic actuator uses the pressure difference between the two sides of a piston to drive the piston rod, thus providing power output in the form of linear reciprocating motion [3–5]. The viscous hydraulic vibration absorber’s working principle is the opposite of this. When external vibration energy pushes the piston rod to make the piston reciprocate in the hydraulic cylinder, the hydraulic oil in the cylinder flows through a preset orifice. Internal friction of the hydraulic oil and wall friction between the piston and the inner wall of the cylinder convert the mechanical energy of the vibration into heat energy, which is dissipated to achieve vibration absorption [6]. Depending on the various designs of hydraulic circuits and components, the hydraulic cylinders present different operating performance and characteristics.

In order to improve the durability and efficiency of a hydraulic pump for internal oil supply to a hydraulic cylinder at low speeds, Zagar et al. proposed the control concept of digital valves and proved that it can avoid adverse hydraulic pump operating conditions [7]. Berikbaeva et al. used a combination of cutting and surface plastic deformation to perform deep hole processing, thereby improving the durability of hydraulic cylinders [8]. Padovani et al. proposed an electro-hydraulic self-contained single-rod cylinder system with passive load holding and energy recovering capability, and verified the correctness of the proposed dynamic model and system performance through bench tests [9]. It can be concluded from the recent literature that scholars are trying to optimize the durability and performance of the hydraulic cylinder system by changing the processing, design, control method, etc. [10, 11] It can also be concluded that the sealing unit in the hydraulic cylinder system is closely related to the transmission efficiency and durability of hydraulic cylinders [12–14].

O-rings are widely used in the design of sealing systems for hydraulic and pneumatic machinery due to their high pressure resistance, reliable function, low price, simple manufacturing and simple installation requirements [15, 16]. The hydraulic cylinder is a dynamic system with reciprocating motion. Generally, a sealing groove is made on the piston to install O-rings for sealing to isolate the cavities present on both sides of the piston. Therefore, occurrence of friction between the O-ring and the inner wall of the cylinder is inevitable [17]. Yanada et al. studied the friction under sliding regime through a series of comparison experiments. This work denied the accuracy of the LuGre model and proposed an improved model based on the LuGre model. The improved model was shown to have higher accuracy in reflecting the real friction behavior in the hydraulic mechanism [18]. Pan et al. studied the friction behavior by using different forms of sealing rings in double-acting hydraulic actuators. The dynamic friction force under different velocity and acceleration conditions was compared and analyzed [19]. Azzi et al. investigated the friction behavior of the seals installed on the piston and piston rod. They reported that pneumatic pressure has greater influence on friction than on velocity, and that the friction of piston seals accounted for more than 90% of the total friction in the cylinder system [20]. Thus, friction in hydraulic cylinder systems has been extensively studied, and is generally regarded to have a negative effect [20–23]. Therefore, scholars have tried to minimize friction without weakening the sealing performance by studying its dynamic and steady-state behavior.

On the other hand, Qin et al. proposed a novel design of a vibration energy absorbing mechanism. They proposed that the friction force occurring at the piston sealing unit in the hydraulic cylinder can be limited to a certain range by controlling the machining accuracy of metal components, so as to realize the automatic adjustment between their two vibration energy absorbing modules [24]. In order to make reasonable use of this friction force, a test bench for observing the sliding friction characteristics in the hydraulic cylinder system was built in this study, and the prototype tested was manufactured using five-axis precision machining and measurement. The friction characteristics of hydraulic cylinders were tested under different conditions of hardness, inner diameter of piston seal ring groove, reciprocating movement displacement and frequency. Through the integration and comparison of the measured data, they summarized and discussed the relationship between friction behavior and the changes in preset variables.

## Experimental setup

**Fig 1:**
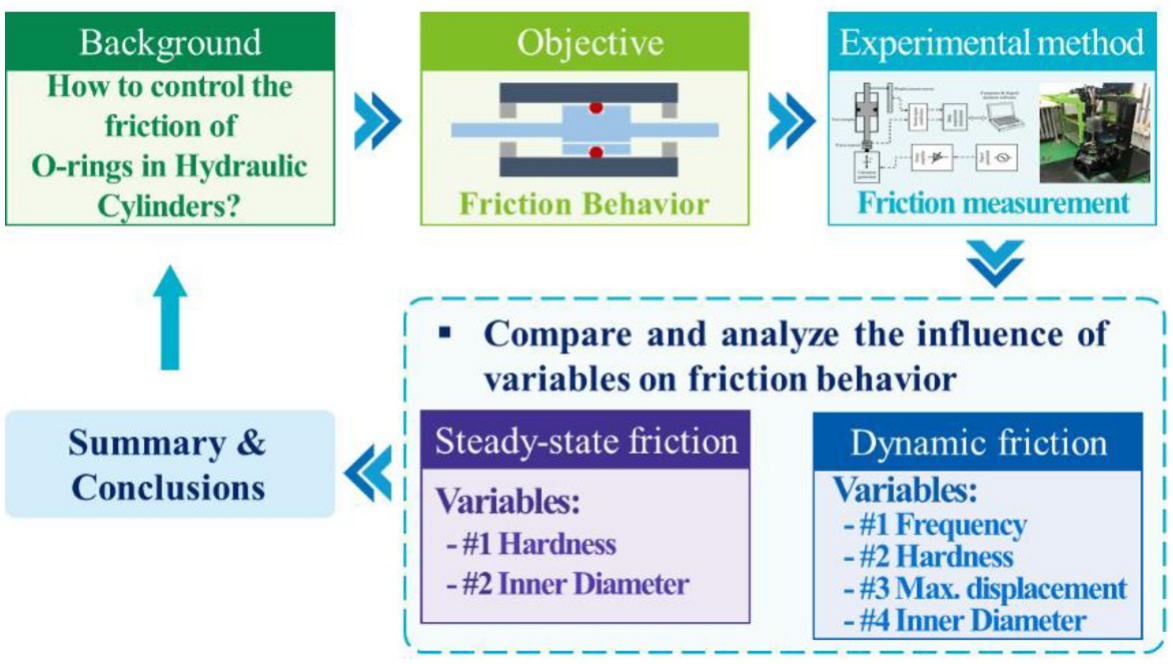
Flowchart of this research work.

Fig 1 depicts the research work’s flowchart. This paper aims to investigate the friction behavior of an O-ring in a hydraulic cylinder using experimental methods in light of the research background mentioned above. To further control the friction force of the O-ring in the hydraulic cylinder, the effects of static friction and dynamic friction on various variables were investigated and the findings were then summarized.

Usually, for friction force measurement inside a hydraulic cylinder system, the electronic control unit and the hydraulic pump are used as the excitation input, a displacement meter is used to measure the displacement, and the signal from the accelerometer and the mass are used to calculate the friction force. In contrast with the conventional method, this study utilized an electronic control unit and an electromagnetic exciter as the excitation input, and a strain-based displacement sensor and a force sensor were used to directly obtain the displacement and force data.

**Fig 2:**
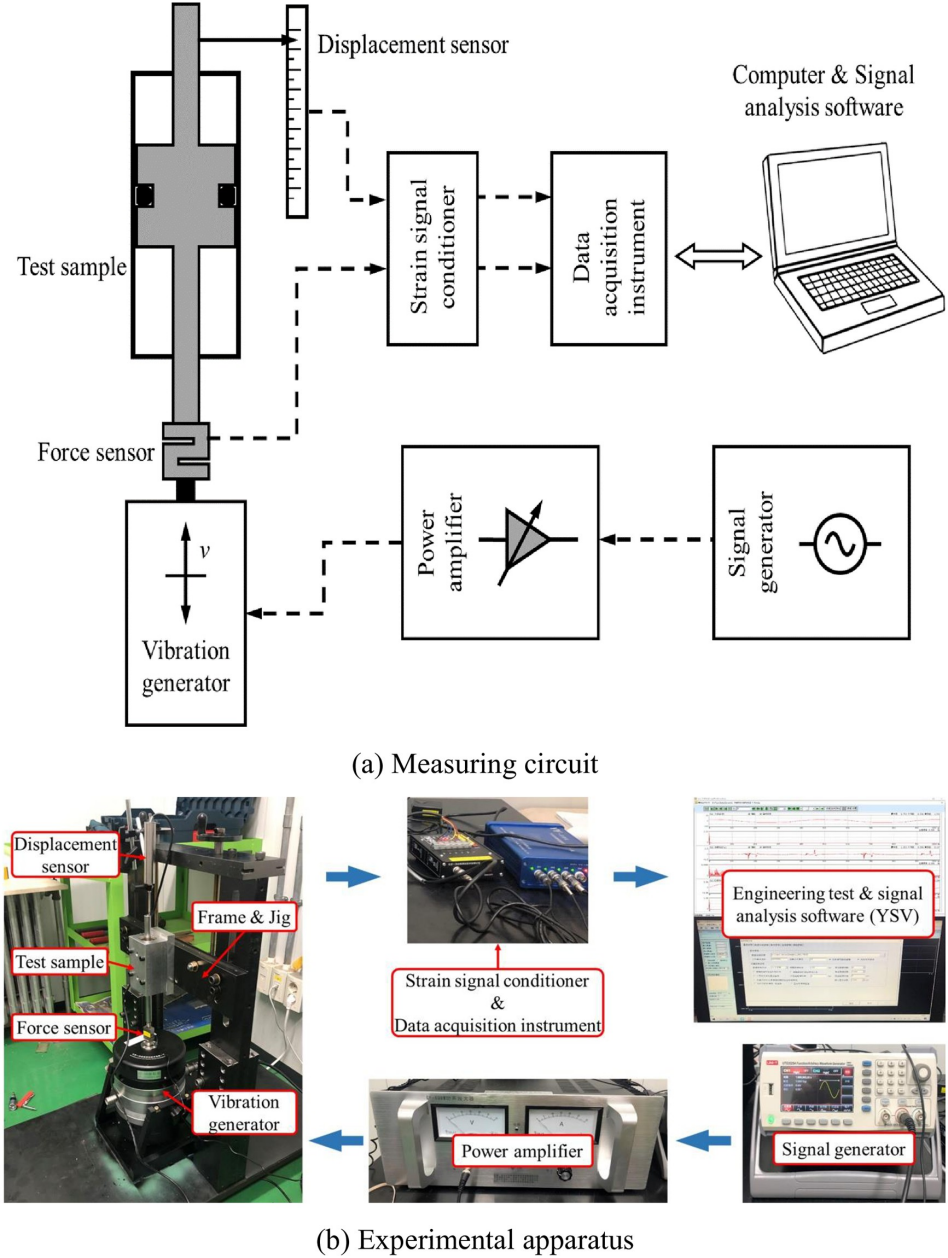
Schematic of the experimental apparatus. (a) Measuring circuit. (b) Experimental apparatus.

The schematic diagram of the friction force measurement system used in this study is shown in Fig 2. The lower part of the figure is the vibration force supply unit and the upper part is the measurement and data analysis unit.

The excitation signal is edited in the signal generator (UTG, 2025A) and then output to the power amplifier (YIYANG, GF-800W). In this study, the saw-tooth waveform is used to measure the steady-state friction under different working conditions, and the sine waveform is used to measure the dynamic friction. Waveform parameters, such as frequency and amplitude (voltage signal), can be selected and edited in the signal generator. The output end of the power amplifier is directly connected to the electromagnetic vibration generator (YIYANG, JZ-50). The gain of the power amplifier is adjustable, and the rated power output is 800W. The vibration generator is rated at 500N maximum excitation force output, and its maximum excitation displacement range is ±12.5mm. In order to reflect the change in friction more accurately, a high-precision strain-based force sensor (YIYANG, YC-1) with a maximum range of 5000N is fixed on the upper end of the mandrel of the vibration generator, and the other end of the force sensor is connected directly to the piston rod of the experimental prototype.

**Fig 3:**
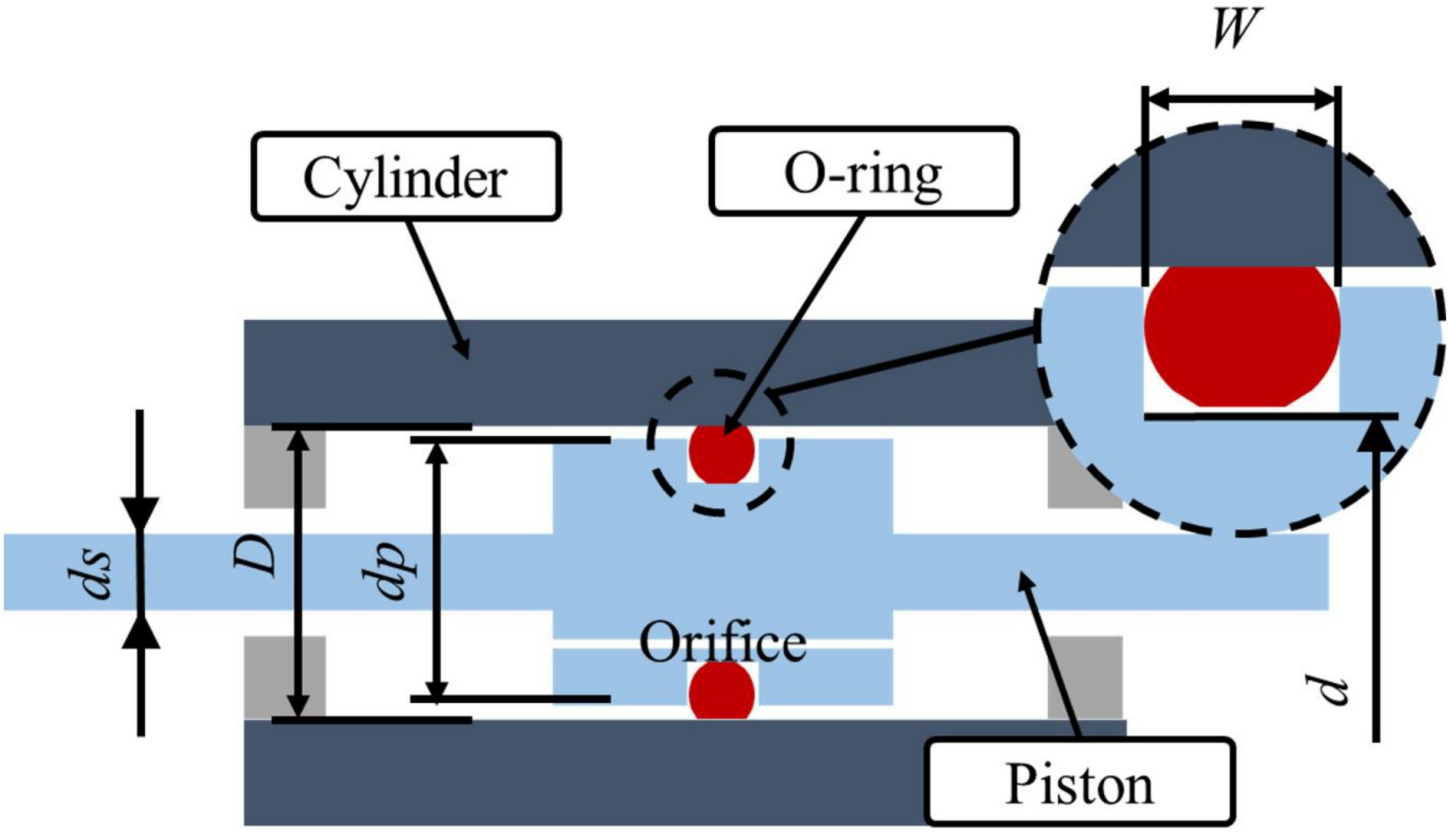
Schematic of the hydraulic cylinder system and its key dimensions.

Fig 3 shows the schematic diagram and key dimensions of the tested hydraulic cylinder prototype. It can be seen that the prototype is composed of three main parts: a cylinder, a piston with an orifice (with a rod connected to it) and an O-ring seal. Taking into account the pressure imbalance caused by the asymmetric piston rod [25–27] and the installation of the sensors, a symmetrical structure of the piston rod is used in this study. The main purpose of this study is to observe the friction behavior of the O-ring, the effects of the hydraulic oil pressure on the O-ring are ignored. As mentioned before, one end of the piston rod is fixed to the top mandrel of the vibration generator, while the O-ring in the middle of the piston limits the radial movement. Therefore, in order to prevent the other frictional forces from affecting the measurement results, the maximum clearance fit design is adopted between the piston rod and the hydraulic cylinder head.

The test frame and jigs are made of steel to increase their natural vibration frequency in order to avoid the occurrence of resonance during the experiment. The test frame was designed precisely to maintain the concentricity between the mandrel of the vibration generator and the piston rod of the prototype. The height of the prototype can be adjusted by the rocker mechanism designed as part of the test frame to ensure availability of enough space for the displacement of the piston in the hydraulic cylinder.

A displacement sensor (YIYANG, YW-50) with a measurement range of 50mm is fixed at the top of the piston rod of the prototype. The force and displacement sensors used in the measurement system are strain-based sensors. In order to convert the strain signal into an electrical signal that can be read by the data acquisition instrument, the sensors are individually connected to the strain conditioner (YIYANG, YSV7001), and the output of the conditioner is connected to the data acquisition instrument (YIYANG, YSV8004). Finally, the data signal from the acquisition instrument is transmitted to the signal analysis software (YSV V8.0) running on a computer through an Ethernet connection for visualization, recording and data analysis. Table 1 shows the basic information of the main instruments.

**Table 1:** Main instruments used in the experimental setup.

No.	Instrument	Mark	Specification
1	JZ-50 vibration generator	YIYANG	Frequency range: DC~4000Hz, Force rated(Peak): 500N, Input current: 25A, Displacement: ± 12.5mm
2	GF-800W power amplifier	YIYANG	Rated power output: 800W, Rated voltage output: 30Vrms, Rated current output: 30A, Frequency range: 1Hz-20KHz
3	2025A Signal Generator	UTG	Function/waveform generator
4	YSV Engineering test and signal analysis Software V8.0	YIYANG	Signal sample, storage, analysis
5	YSV8004 24 bit network acquisition instrument	YIYANG	24 bit Δ-Σ A/D conversion chip, 4 channels, 51.2ks/s
6	YW-50 strain displacement sensor	YIYANG	Range: 50mm; Resistance: 350Ω; Frequency response: 0-30Hz
7	YSV7001 Strain Signal Conditioner	YIYANG	Frequency response: DC-10KHz, Gain:100; Bridge voltage: 2Vor5V
8	SFSAX Strain Force Sensor	YIYANG	Strain gauge; Range: 200kgf; Resistance: 350Ω

## Measurement results and discussion

The key parameters and variables of the prototype used in this study are given in Tables 2 and 3. It can be seen that there are two main variables in this study: (1) the inner diameter d of the groove of the sealing ring and (2) the hardness of the O-ring. The purpose of this research is to compare and observe the O-ring’s friction behavior under different conditions, in order to summarize the related rules, so that in future work involving O-rings, the design process to find a reasonable friction value can be sped up.

**Table 2:** Key design parameters of the prototype.

No.	*D* (mm)	*dp* (mm)	*ds* (mm)	*W* (mm)	*d* (mm)
1	40.05	39.90	16.00	5.00	34.59
2					34.10
3					33.75

**Table 3:** Parameters of the O-ring.

No.	Dimension Code	Material	Hardness (SH.A)
1	NOK-A402	NBR	60
2			70
3			90

### Steady-state friction

The general friction force expression of a hydraulic cylinder can be expressed as follows:

$$F_{r}=p_{1}A_{1}-p_{2}A_{2}+mg+ma$$ (1)

where, *p*_1_*A*_1_ − *p*_2_*A*_2_ represents the hydraulic force difference acting on the piston due to the liquid in the hydraulic cylinder. *mg* and *ma* respectively represent the weight of the piston and its acceleration force. However, as already mentioned, the effect of the hydraulic pressure is not considered in this study. In addition, the strain-based force sensor can filter the influence of its own weight to directly measure the acceleration force. Therefore, for the experimental system built in this study, the force data measured during the piston movement at a constant speed (no acceleration) represents the steady-state friction force generated by the O-ring and the cylinder wall.

#### Varying the hardness of O-rings

In order to compare the characteristics of the steady-state friction behavior of O-rings made with the same material but with different hardness values, we carried out the friction measurement tests with three natural rubber O-rings with hardness values of 60SH.A, 70SH.A and 90SH.A. To obtain the steady-state friction, the saw-tooth waveform was applied as the input signal. This ensured that the piston always had a uniform motion during its reciprocating movement. Before starting to record the data, the power amplifier and displacement signal tuned to limit the displacement to 5.0mm, so as to ensure the consistency of the measured steady-state friction data. This also ensured that the obtained data was not affected by the difference in displacements.

**Fig 4:**
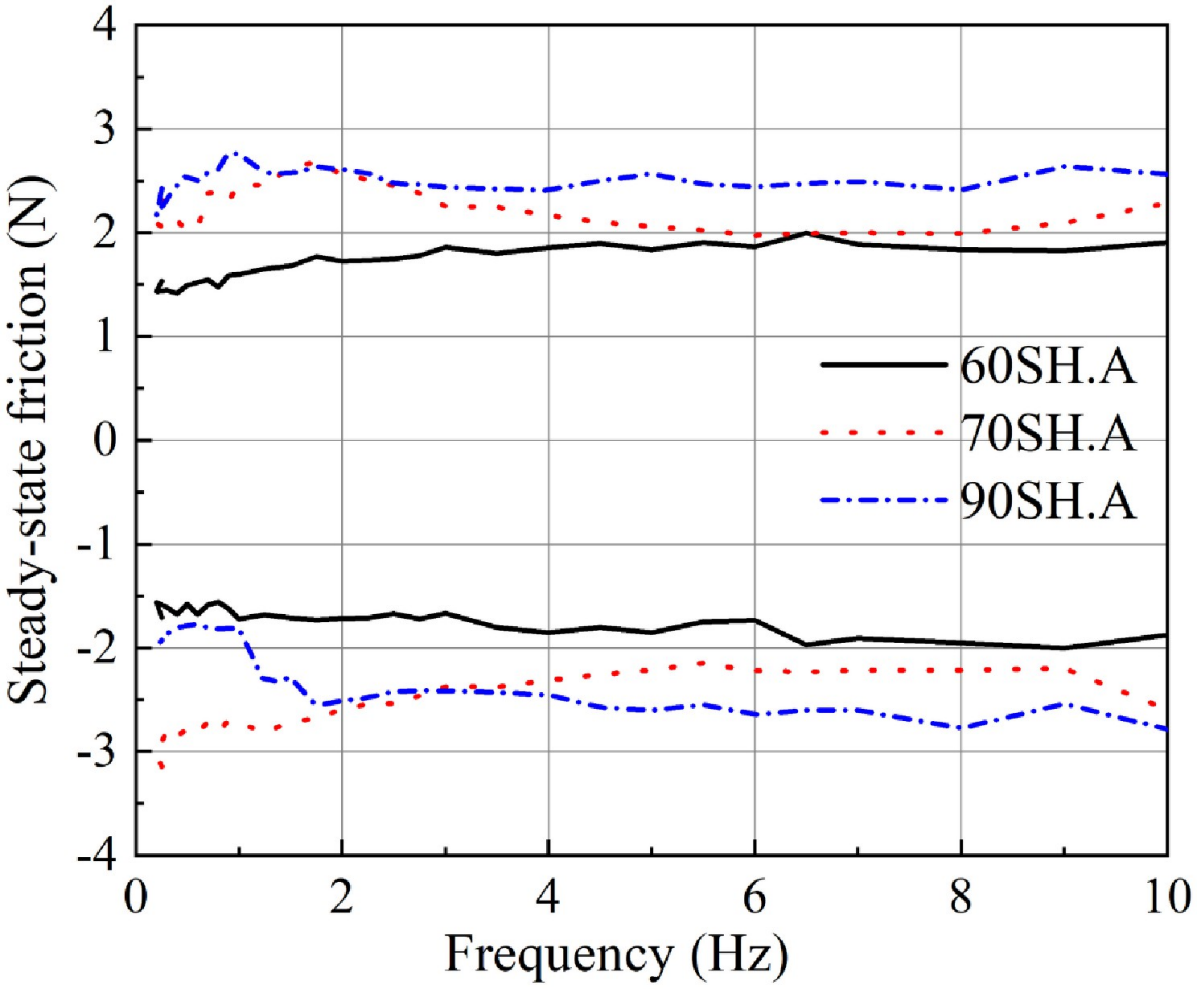
Steady-state friction forces recorded with O-rings of different hardness during reciprocating movements with different frequencies.

Fig 4 shows the results of steady-state friction measurement at different frequencies for the three O-rings with different hardness values. The black continuous line represents the results for the O-ring with a hardness of 60SH.A. The red dotted line and the blue dash-dotted line respectively represent the measurement results of the O-rings with the hardness values of 70SH.A and 90SH.A.

It can be seen that the O-ring with a hardness value of 60SH.A exhibits a relatively small steady-state friction force of approximately 1.5N at the low frequency (below 1Hz), during both forward and backward directed movements. Increase in the frequency of reciprocating motion results in a slight increase in the steady-state friction. However, the steady-state friction force of the O-ring with a hardness value of 60SH.A stayed below 2N during both forward and backward directed movements happening at less than 10Hz.

From the results observed during the forward directed movements of the O-ring with a hardness value of 70SH.A, it can be seen that the friction force has a maximum value of 2.6N at 1.7 Hz. Beyond that, there was a slight decreasing trend up till 6.5Hz. Between 6.5Hz and 8Hz, there was no obvious change, but between 8Hz and 10Hz, a slight increase was observed. The results observed during the backward directed movement revealed that the steady-state friction force was close to 3N in the low frequency region (below 1.7Hz). Beyond this frequency, the results showed trends similar to those observed with the forward directed movement.

Results with the O-ring of 90SH.A hardness revealed that during both forward and backward directed movements the friction has a relatively smaller starting value, which then increases obviously up till 1.7Hz. Beyond 1.7 Hz, the frictional force showed an even increase with the change in frequency. Overall, the steady-state friction of the entire process did not exceed 2.8N.

#### Varying the inner diameter of O-ring grooves

In order to explore the influence of different O-ring groove inner diameters on the steady-state friction of the system, in this study, precision machining technology was used to produce three piston prototypes with O-ring groove inner diameters of 34.59mm, 34.10mm and 33.75mm. The friction generated by these different diameters was tested using the same process as that explained in the previous section. The O-ring used in these tests had a hardness of 90SH.A.

**Fig 5:**
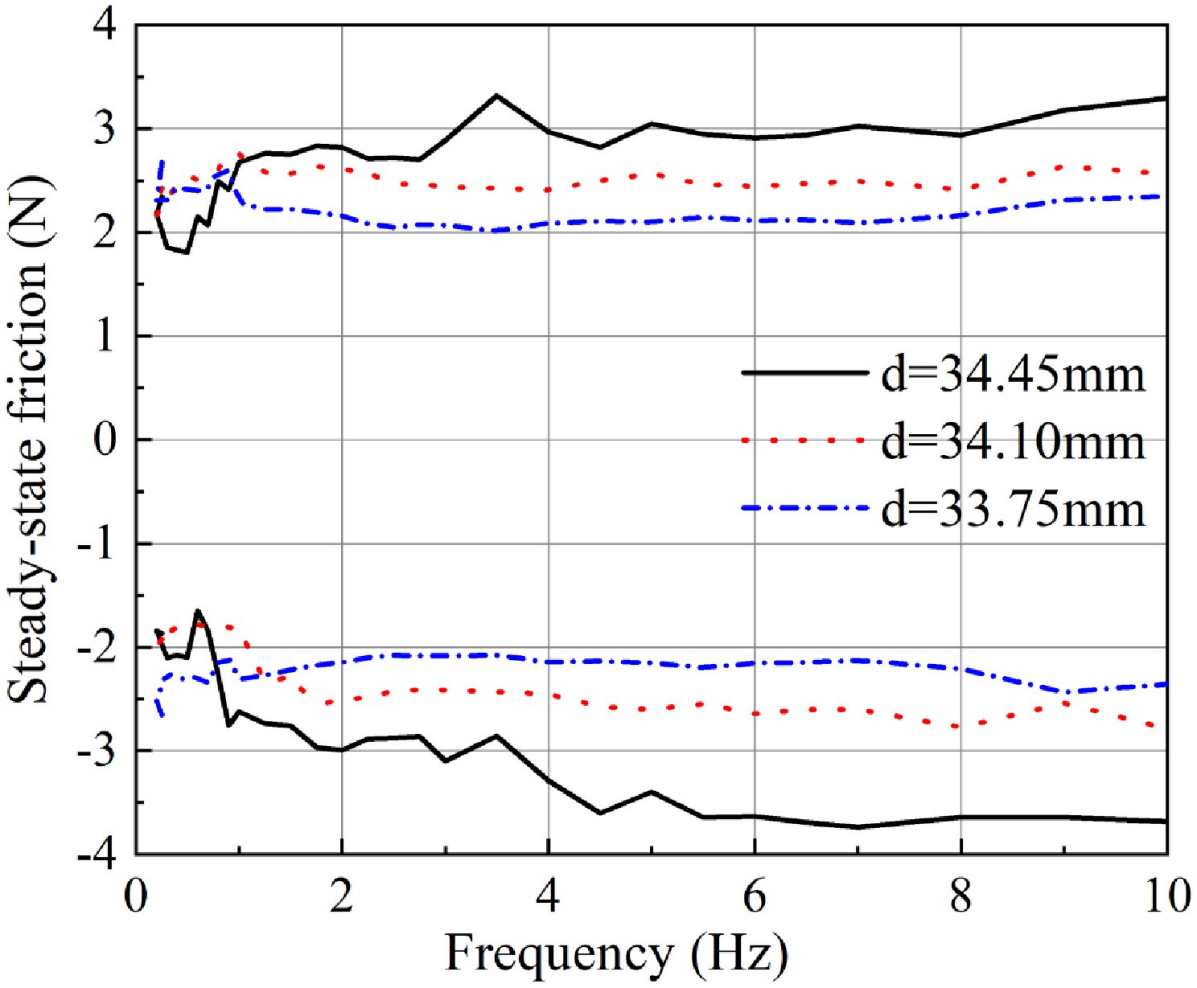
Steady-state friction force with O-ring grooves of different inner diameters under different frequency conditions.

The steady-state friction forces observed with the three different groove inner diameters at various frequencies are summarized and compared in Fig 5. The continuous, dotted and dash-dotted lines respectively represent the data recorded for the O-ring grooves with inner diameters of 34.59mm, 34.10mm and 33.75mm.

It can be seen from the results that the three different groove inner diameters exhibit small steady-state friction forces in the low-frequency region (under 1Hz). Between 1Hz and 6Hz, the steady-state friction force of d = 34.45mm shows an obvious surge where the friction force increased from about 2.0N to nearly 3.7N.

The steady-state friction force of the groove with inner diameter of d = 34.10mm also showed a small increase in this frequency range. However, the steady-state friction force of the O-ring groove with inner diameter of d = 33.75mm remained relatively stable in the entire frequency range and did not show any obvious change.

The overall results are as we expected, i.e. the smaller the groove inner diameter, the smaller the steady-state friction force. This is because smaller inner diameter of the groove means a smaller fill factor of the O-ring in the cross section, which results in smaller extrusion deformation of the O-ring. This reduces the normal pressure between the O-ring and the cylinder, which reduces the friction force but also weakens the sealing effect of the O-ring. However, a small change in the O-ring groove inner diameter within a certain range is acceptable.

### Dynamic friction

**Fig 6:**
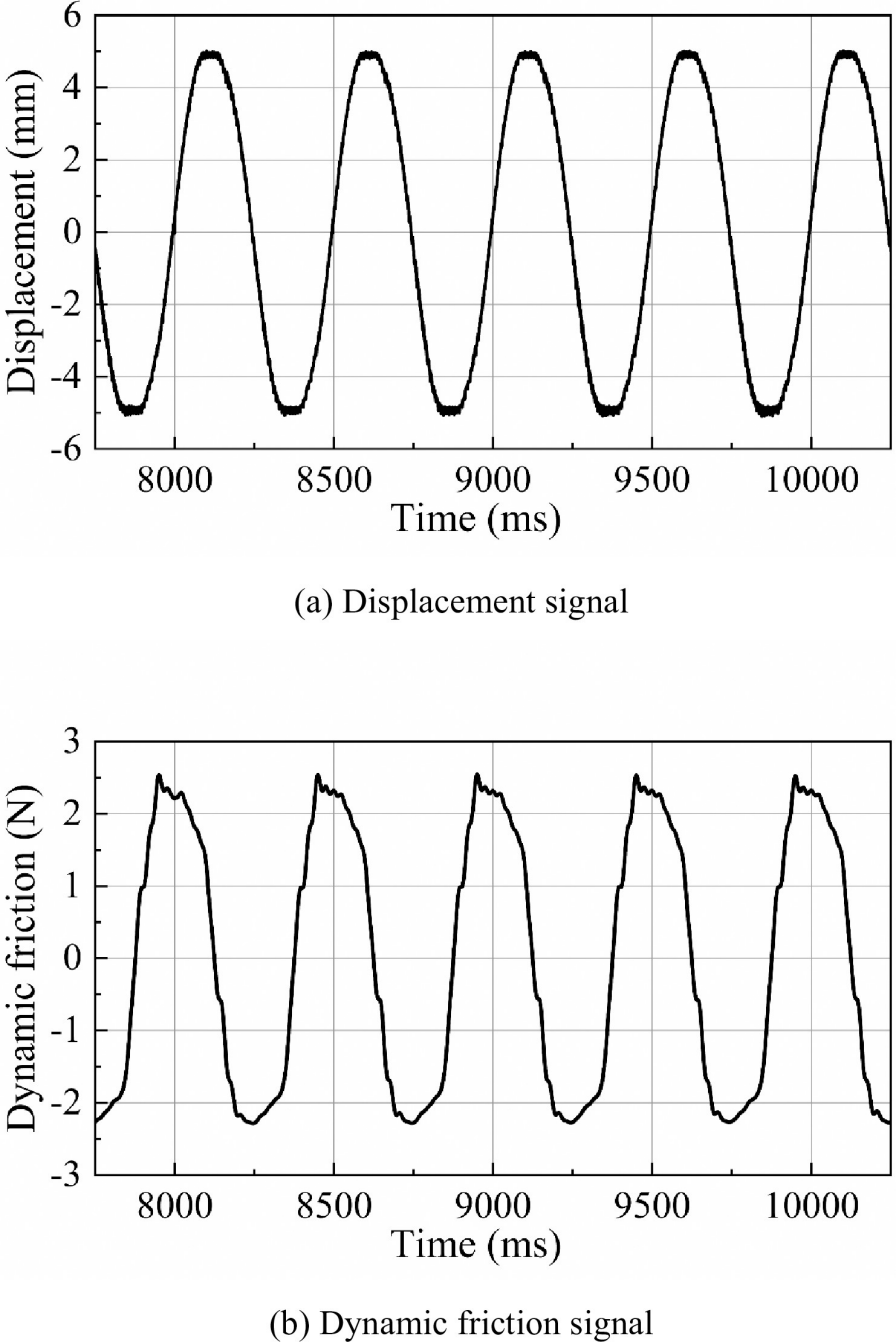
Example data of dynamic friction measurement. (a) Displacement signal. (b) Dynamic friction signal.

The dynamic friction force is also tested and analyzed using the same measurement system as that used for the steady-state friction measurement. However, to measure the dynamic friction force, the input used is a sine signal instead of a saw-tooth signal. Fig 6 shows an example of the displacement and friction signals recorded during a dynamic friction test. Fig 6(a) shows the measured displacement signal. It can be seen that the input from the signal generator after power amplification is well presented on the piston rod of the prototype. Fig 6(b) presents the force signal measured by the strain-based force sensor. Similar to the steady-state friction tests, the dynamic friction behaviors of three O-rings with different hardness values and three different O-ring groove inner diameters are recorded and compared in this section.

#### Varying the hardness of O-rings

**Fig 7:**
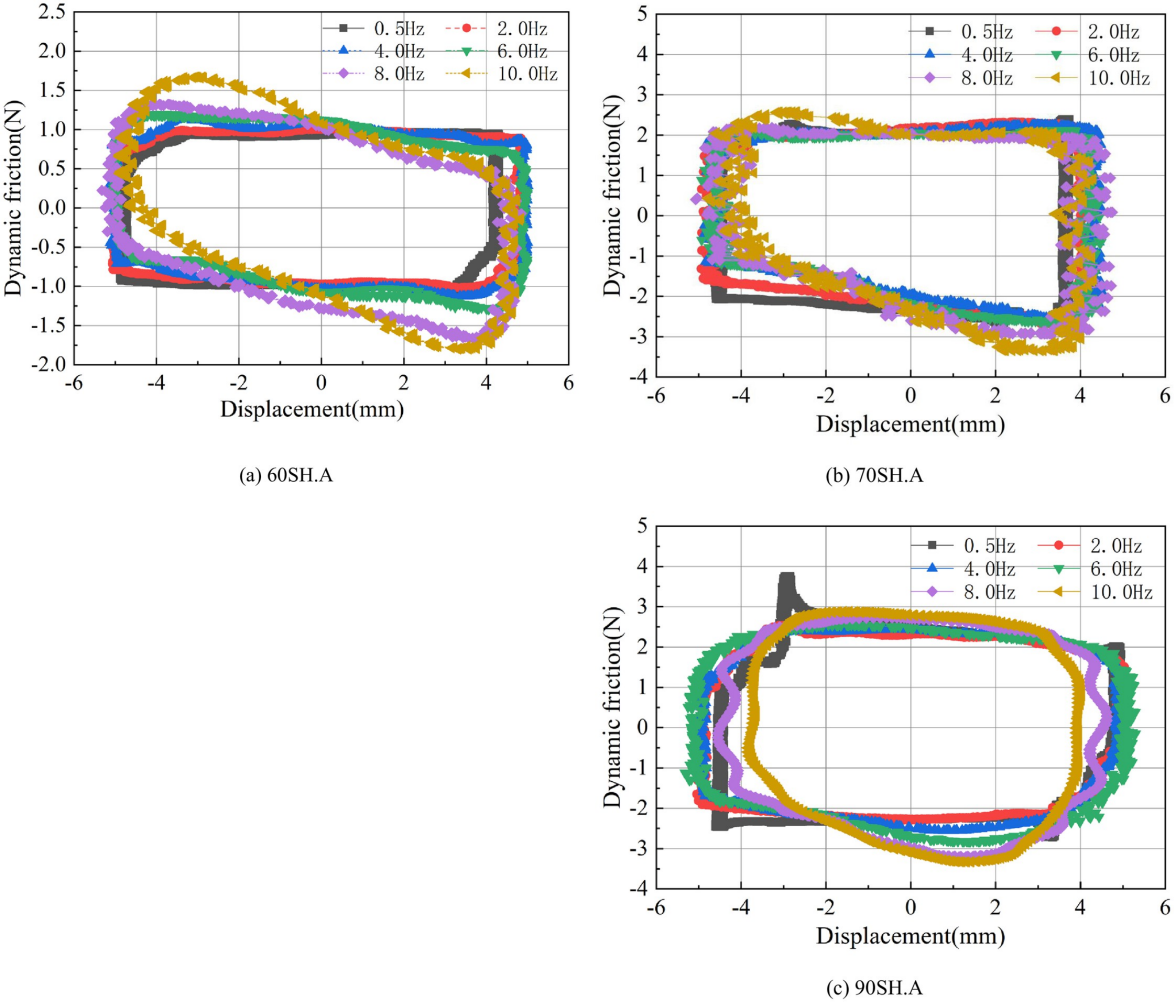
Dynamic friction forces of O-rings with different hardness values vs. piston displacements at different reciprocating frequencies. (a) 60SH.A. (b) 70SH.A. (c) 90SH.A.

Fig 7 shows the variation of dynamic friction of O-rings with three different hardness values at different reciprocating frequencies.

**Fig 8:**
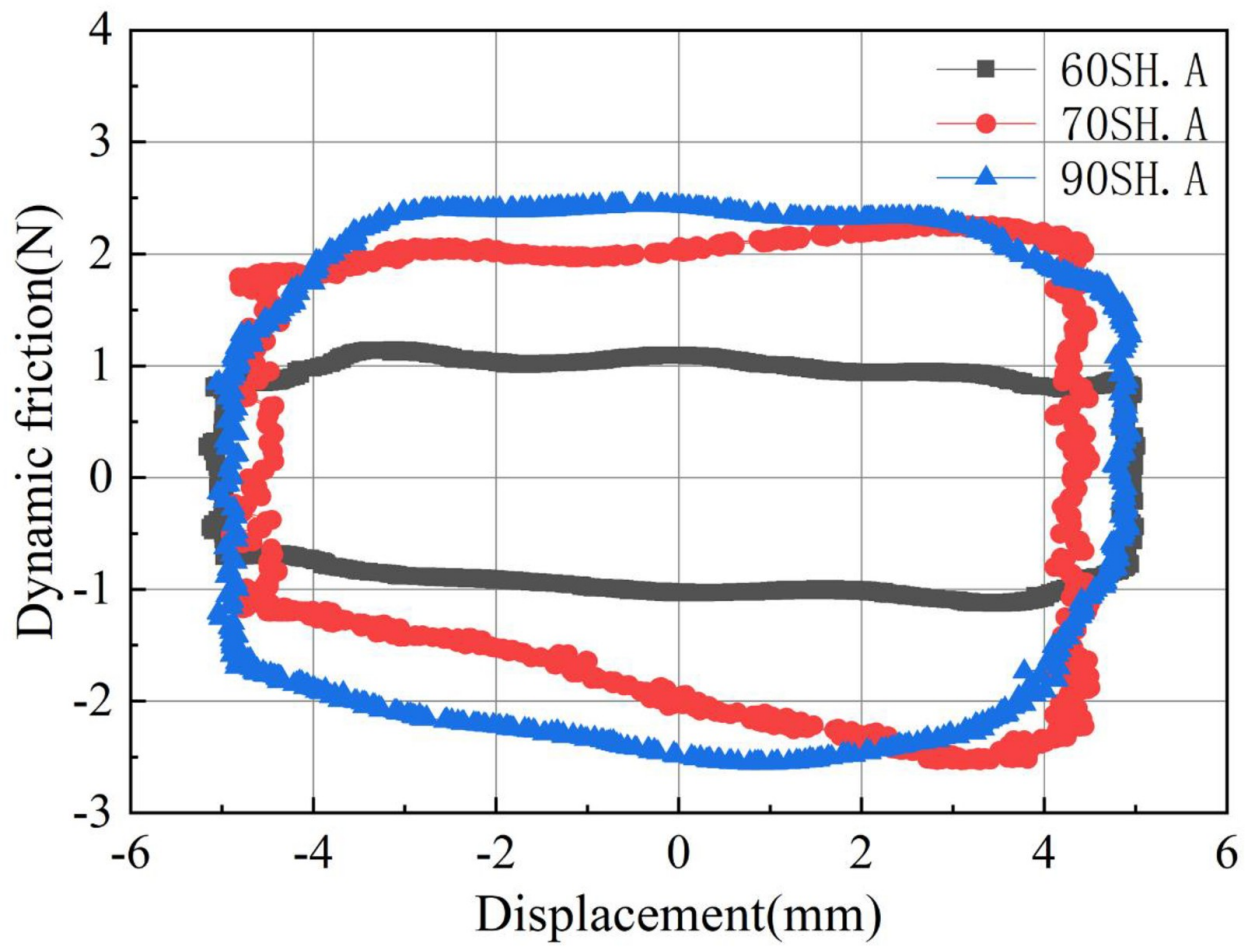
Dynamic friction force with O-rings of different hardness values vs. piston displacement at 4.0Hz reciprocating frequency.

**Fig 9:**
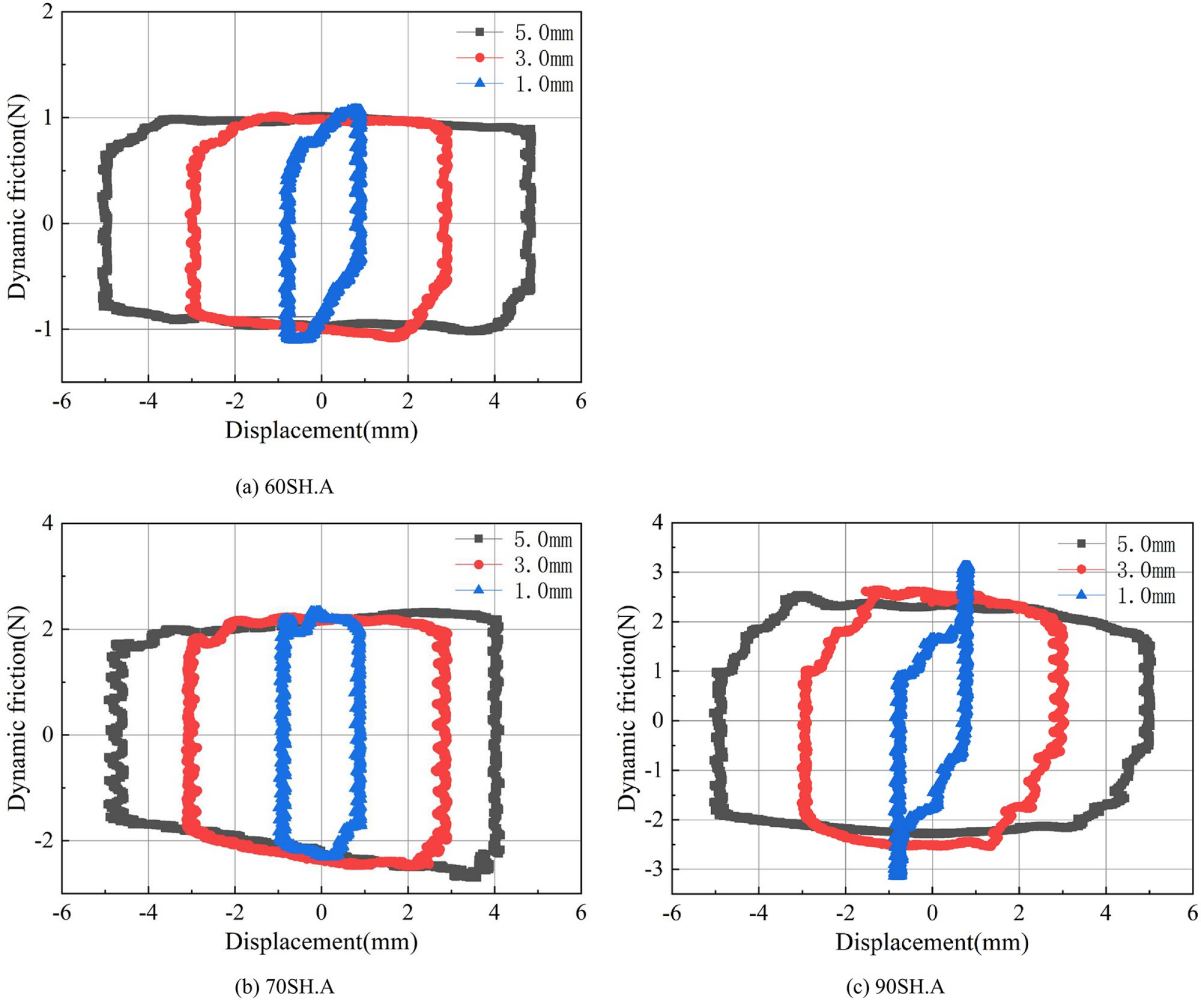
Dynamic friction forces recorded for O-rings with different hardness values under different maximum displacement conditions at 2.0Hz reciprocating frequency. (a) 60SH.A. (b) 70SH.A. (c) 90SH.A.

**Fig 10:**
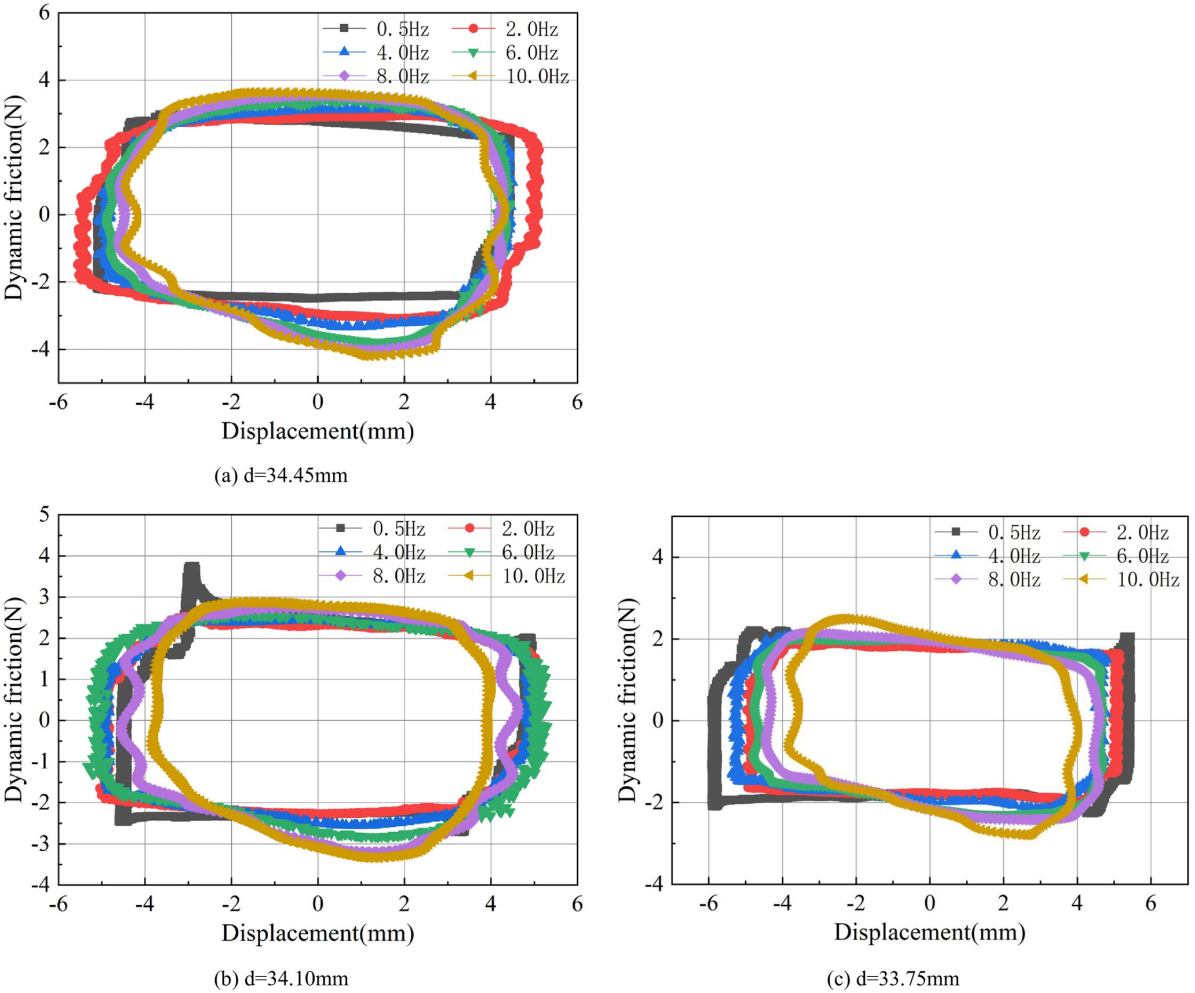
Dynamic friction force with different O-ring groove inner diameters vs. piston displacement at different reciprocating frequencies. (a) d = 34.45mm. (b) d = 34.10mm. (c) d = 33.75mm.

**Fig 11:**
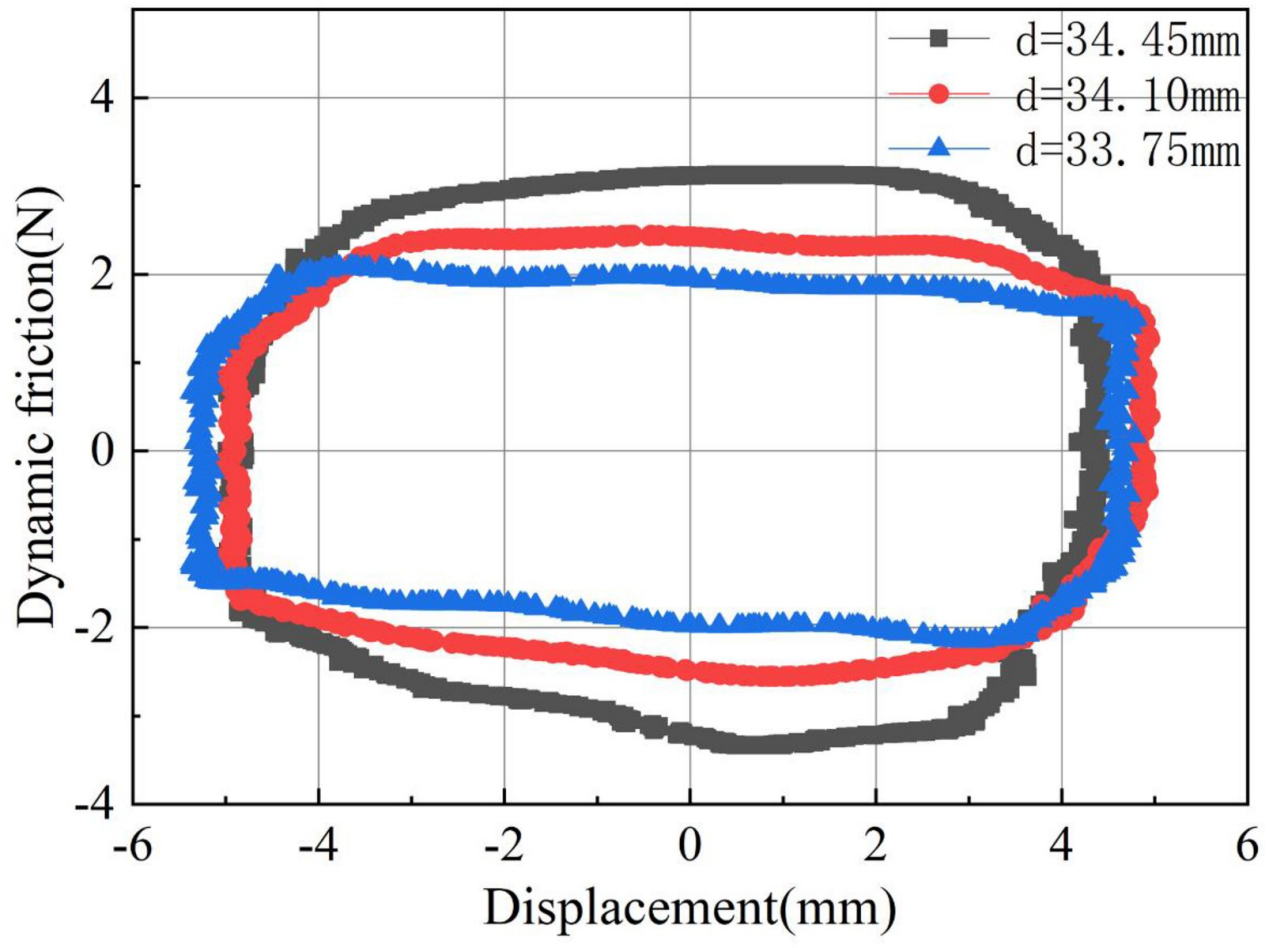
Dynamic friction force with different O-ring groove inner diameters vs. piston displacement at 4.0Hz reciprocating frequency.

**Fig 12:**
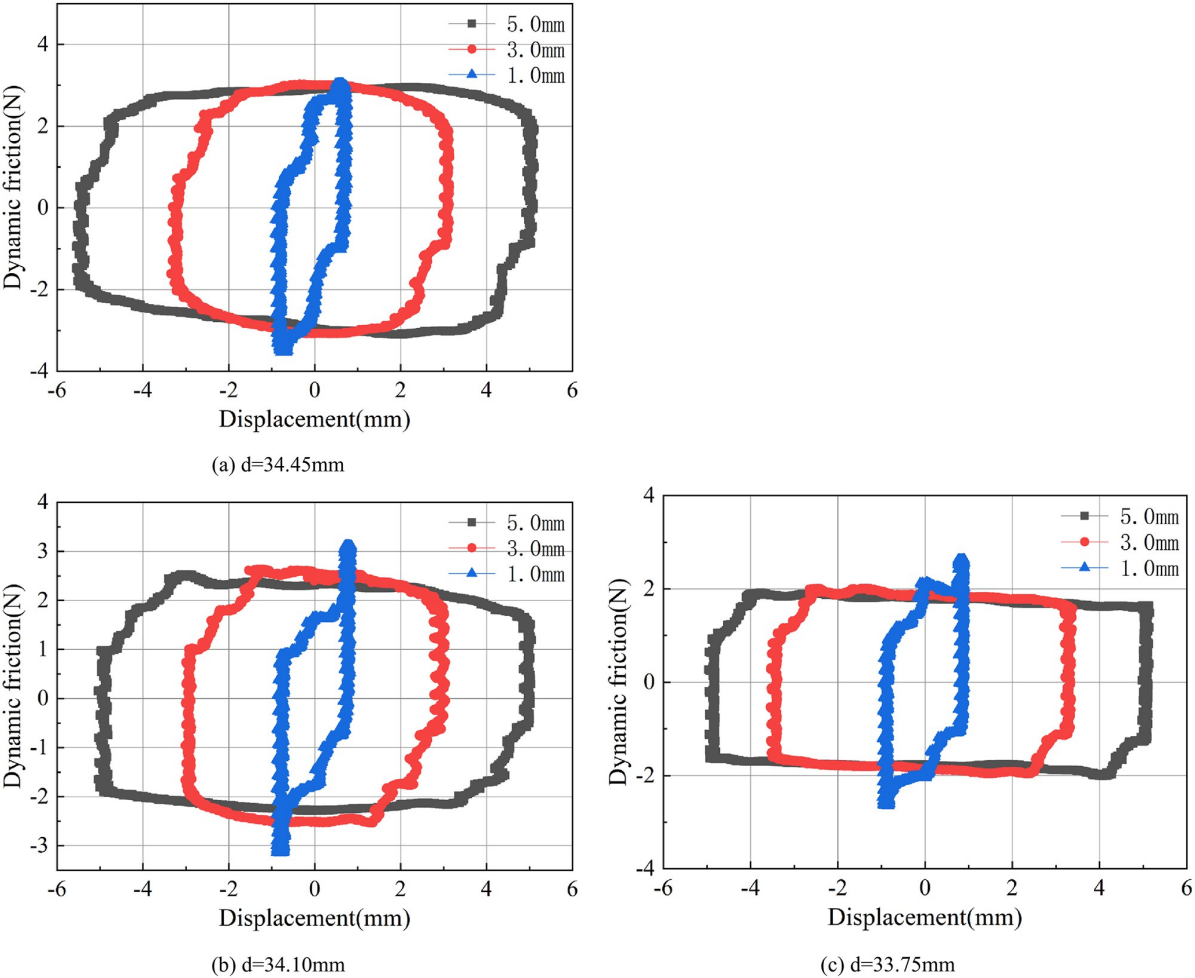
Dynamic friction force with different O-ring groove inner diameters under different maximum piston displacements at 2.0Hz reciprocating frequency. (a) d = 34.45mm. (b) d = 34.10mm. (c) d = 33.75mm.

When an O-ring with a hardness of 60SH.A is installed, as can be seen in Fig 7(a). The maximum positive dynamic frictional force appears when the reverse stroke ends and the forward stroke starts, and the frictional force value decreases with the increase in displacement. The dynamic friction at the reverse stroke shows a similar behavior. When the reverse motion begins, the largest negative friction force appears, which then decreases continuously through the entire stroke. Thus, the curves plotting the dynamic friction force take the shape of parallelograms, as shown in Figs 7–12. The curves with different colors and marks indicate different reciprocating frequencies. It can be seen that the greater the reciprocating frequency, the smaller the acute angle of the parallelogram. This means that the maximum value of positive and negative dynamic friction increases and the minimum value decreases with the increase in the reciprocating frequency.

Fig 7(b) shows the dynamic friction results of the O-ring with a hardness of 70SH.A at different reciprocating frequencies. Comparing with the friction results of the O-ring with a hardness of 60SH.A, shown in Fig 7(a), there is a similar reduction in friction during the reverse stroke. However, the friction force at the beginning of the forward stroke at low frequency is slightly less than the maximum friction force at the end of the forward stroke. As the frequency increases, the positive friction at the starting point of the forward stroke increases. In high-frequency motion (10.0 Hz), the maximum friction force appears at the starting point of the forward stroke, and it decreases as the displacement increases.

Fig 7(c) presents the friction behavior when the O-ring with a hardness of 90SH.A is used. Under the reciprocating frequency of 0.5 Hz, the maximum value of dynamic friction appeared at the start of the forward stroke, which was followed by a sharp decrease. A gradual decrease in the friction force is observed during the rear curve of the forward stroke, which is similar to the abovementioned behavior observed with the other O-rings. The results at other reciprocating frequencies are similar to those recorded for the O-ring with a hardness of 60SH.A.

In order to more intuitively observe the difference in dynamic friction caused by O-rings with different hardness values, their dynamic friction behavior curves at 4.0Hz are summarized as shown in Fig 8. It can be seen that the greater the hardness, the greater the maximum value of dynamic friction during the stroke, and the larger the area enclosed by the curve. It is worth emphasizing here that the area enclosed by the curve is the energy consumed due to the friction of the O-ring.

Fig 9 shows the friction behavior of the O-rings with different hardness under different maximum displacements. All the data shown was recorded at 2.0Hz reciprocating frequency. As expected, the energy consumption area increases with increase in the maximum stroke displacement. However, it can be observed that the O-rings with the hardness values of 60SH.A and 90SH.A show a sudden increase in the maximum friction force at the ends of both the strokes when the maximum displacement is ±1.0mm. It can also be observed through the comparison of these three results that as the O-ring hardness increases, the average value of dynamic friction during the motion also increases.

#### Varying the inner diameter of O-ring grooves

Precision machining technology has developed rapidly in recent years and now the precision of machining can be accurately controlled within the error range of only 0.1~1.0μm [28–31]. This research has attempted to explore the friction behavior between the sealing ring and the cylinder through precision machining technology.

Fig 10(a)–10(c) respectively show the friction behavior of three O-ring grooves with inner diameters of 34.45mm, 34.10mm and 33.75mm at different reciprocating frequencies. In order to facilitate comparison, all other parameters are kept constant and the O-ring with a hardness of 90SH.A is used in all tests. By observing each figure, it can be concluded that similar to the results shown in Fig 7, the dynamic frictional force at the starting point of the stroke in both the forward and reverse directions increases as the frequency of reciprocating motion increases, and then decreases with the change in the displacement. Furthermore, during the changing of stroke direction, it was observed that the sudden change of the dynamic friction caused the displacement to show a slight fluctuation. Compared with the other curves, the curve of d = 33.75mm is more angular, and as the inner diameter of the groove increases, the energy consumption area also increases while the angularity of the curve gradually disappears.

Similarly, the O-ring friction curves with different groove inner diameters at a reciprocating frequency of 4.0Hz are summarized in Fig 11. It can be seen that a larger groove inner diameter increases the maximum friction force of the O-ring during each stroke movement, thereby increasing the energy consumption area of the entire process.

Fig 12 shows the dynamic friction force curves of the O-rings installed in grooves with different inner diameters under different maximum displacements. The reciprocating frequency was fixed at 2.0Hz. Similar to the results with O-rings of different hardness values, the energy consumption area increases as the maximum stroke displacement is increased. When the maximum stroke is set as ±3.0mm and ±5.0mm, the dynamic friction changes are smooth in both the forward and reverse directions. However, when the maximum stroke is set to ±1.0mm, there is a sharp increase in dynamic friction at the ends of both forward and reverse strokes, which can be attributed to the sudden drop in speed within a short distance.

## Summary and conclusions

In order to observe the friction behavior of the piston seal in a hydraulic cylinder, an experimental setup that can be used to measure both the static and the dynamic friction was established in this research. Under the test conditions consisting of various reciprocating frequencies, maximum stroke displacements and other parameters, the friction behavior of O-rings with different hardness values and of different O-ring groove inner diameters was studied experimentally. After data collection and a series of comparative analyses, we have reached the following conclusions:

1. The steady-state friction force of natural rubber O-rings with different hardness values was tested and measured under different reciprocating frequencies. In the entire frequency domain distribution, it can be seen that the smaller the hardness, the smaller the steady-state friction between the O-ring and the wall of the hydraulic cylinder. However, in the low frequency region of less than 1.7 Hz, there were differences in the steady-state friction of the three O-rings with different hardness values. In future research, it is necessary to use more precise measurements to carry out in depth investigations of the behavior of static friction at low frequencies.
2. The O-ring with a hardness of 90SH.A was installed on pistons with different groove inner diameters manufactured using precision machining technology to measure the steady-state friction. The distribution of steady-state friction in the entire frequency domain revealed that the larger the groove inner diameter, the greater the steady-state friction between the O-ring and the cylinder wall. This is due to the influence of the diameter change on the fill factor of the O-ring in the cross section. In other words, the change in the groove inner diameter changes the deformation of the O-ring, which affects the normal pressure between the O-ring and the cylinder wall. Similar to the results with varying O-ring hardness, the steady-state friction force distribution with varying groove inner diameters in the low-frequency region is also relatively disordered.
3. By comparing the dynamic friction behavior of O-rings with different hardness values at different reciprocating frequencies, it was observed that the greater the reciprocating frequency, the greater the friction force at the starting point of the stroke, which gradually decreases with the change in displacement. However, in the resulting curve of the O-ring with a hardness of 90SH.A, an abnormal starting peak appears at the low frequency of 0.5Hz. It can be observed from the dynamic friction curve at 4.0Hz that under the same maximum displacement condition, the greater the hardness of the O-ring, the greater the average dynamic friction force during the stroke, which means that the frictional energy dissipation effect is more obvious. Comparison of the results with different maximum displacements revealed that under the maximum stroke of ±1.0mm, there is a sudden increase in friction at the end of each stroke.
4. The O-ring with a hardness of 90SH.A was installed on pistons with different groove inner diameters to explore their effect on the dynamic friction behavior. Similar to the results with varying O-ring hardness, the dynamic friction results with varying groove diameters also showed that the higher the reciprocating frequency, the higher the maximum frictional force at the start of each stroke, which gradually decreases as the displacement changes. It was also observed that the larger the inner diameter of the groove, the larger the energy consumption area. The reason of this phenomenon, similar to O-rings with different hardness values, is that both these variables affect the normal pressure between the O-ring and the cylinder wall, which in turn affects the friction behavior.

## Supporting information

S1 Data (ZIP)

## Abbreviations

a: Acceleration of piston movement
*A*_1_: Area under pressure (*p*_1_) on one side
*A*_2_: Area under pressure (*p*_2_) on other side
d: Diameter of O-ring groove bottom
D: Inner diameter of hydraulic cylinder
dp: Outer diameter of piston
ds: Outer diameter of piston shaft
g: Gravitational acceleration
m: Mass of piston
NBR: Nitrile Butadiene Rubber
*p*_1_: Hydraulic pressure on one side of the piston
*p*_2_: Hydraulic pressure on other side of the piston
W: Width of O-ring groove
